# Xanthogranuloma disseminatum: Successfully treated with statins followed by upadacitinib

**DOI:** 10.1016/j.jdcr.2026.01.025

**Published:** 2026-01-28

**Authors:** Fyona Okundia, Iman Bouchelkia, Muna Shakhashiro, Hung Q. Doan, Stephen Tyring

**Affiliations:** a Center for Clinical Studies, Houston, Texas; b The University of Houston College of Medicine, Houston, Texas; c The University of Texas MD Anderson Cancer Center, The Department of Dermatology, Houston, Texas; d The University of Texas Health Science Center, The Department of Dermatoloy, Houston, Texas

**Keywords:** JAK inhibitor, upadacitinib, xanthogranuloma disseminatum

## Introduction

Xanthogranuloma disseminatum (XD) is a rare mucocutaneous xanthomatous disorder characterized by widespread yellow-brown papules and plaques composed of non-Langerhans histiocytes and lipids within the dermis on histology.^1^ Three clinical variants of XD have been described: the most common persistent, nonprogressive form; a progressive form that may involve internal organ systems such as the central nervous system; and the least common, self-resolving form. XD is typically considered a normolipidemic condition, and treatment options remain limited with variable clinical benefit.^1^^,^^2^

Here, we present a unique case of XD in a patient with an abnormal lipid profile, including severe hypertriglyceridemia, who demonstrated significant clinical improvement with a combination of statin therapy and upadacitinib. To our knowledge, this represents the first reported case of XD successfully treated with upadacitinib, highlighting a potential novel therapeutic approach for this rare and difficult-to-treat condition.

## Case report

**Fig 1:**
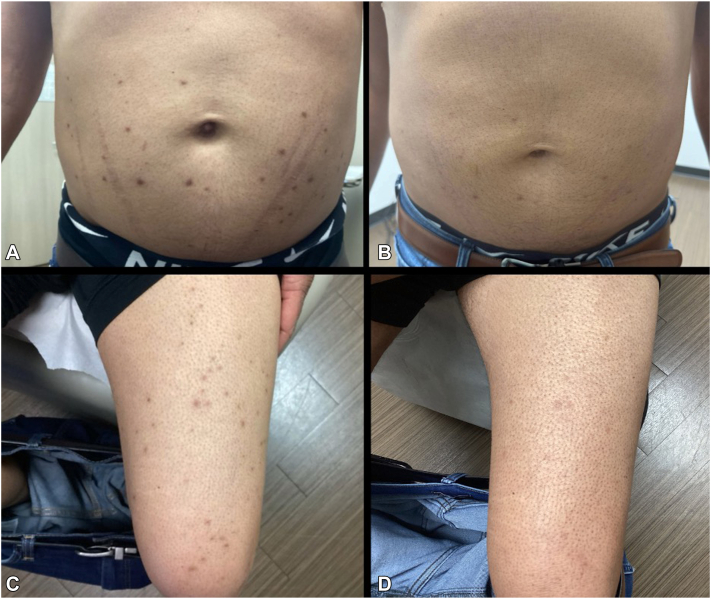
**A,***Yellow-brown papules* on the trunk and **(C)** lower extremity prior to treatment, **(B)** resolving *yellow-brown papules* and plaques on the trunk and **(D)** lower extremity after treatment.

A 38-year-old man presented to the dermatology clinic with a 1 month history of “bumps” widely distributed over his body, which he described as “irritating” and unresponsive to 1% hydrocortisone cream. Physical examination revealed multiple yellow-brown 5 mm papules primarily on his extremities with fewer on his trunk (Fig 1). A shave biopsy was performed. Histopathology revealed a dense diffuse mixed-cell infiltrate in the upper dermis predominantly composed of histocytes, some of which were multinucleate and contained Touton giant cells. This histological and clinical examination was diagnostic for xanthogranuloma disseminatum.

The patient’s initial metabolic profile revealed a glucose of 408 mg/dL, slightly lower sodium and chloride levels and slightly elevated total bilirubin as well as a complete blood count showing an increased white blood cell count with elevated absolute lymphocytes and absolute monocytes. His lipid panel, however, was very abnormal: total cholesterol >1000 mg/dL and triglycerides >10,000 mg/dL.

Initial intervention included simvastatin 40 mg once daily in addition to rosuvastatin 5 mg once daily. After 1 month, his cholesterol level decreased to 184 mg/dL and his triglycerides decreased to 558 mg/dL. After 2 months, his cholesterol level had decreased to 124 mg/dL and his triglycerides had decreased to 191 mg/dL. Cholesterol and triglyceride levels remained under 200 mg/dL for the past year.

Six months after this 99% reduction in his triglycerides, however, the patient had no change in the number or size of his papules. Additionally, no new papules formed after initiating treatment with statins. At that time, upadacitinib 15 mg daily was added to the statins, which resulted in a minor decrease in the size and number of papules. One month later, the dose of Upadacitinib was increased to 30 mg daily with further benefit. Two months after starting Upadacitinib, the dose was increased to 45 mg daily, resulting in complete disappearance of his papules. After 3 months at 45 mg daily, the dose was tapered by 15 mg daily each month and was stopped 7 months after initiation. During the subsequent 6 months, no new papules formed, and the patient discontinued simvastatin and continues rosuvastatin 5 mg daily.

## Discussion

Although XD is traditionally characterized as a normolipidemic disorder, our case demonstrates an atypical presentation of XD accompanied by severe hypertriglyceridemia. Despite aggressive lipid-lowering therapy restoring the patient's cholesterol and triglyceride levels, his papules remained unchanged, showing that lipid control alone cannot adequately address cutaneous disease activity. Conventional therapies for XD, including corticosteroids, immunosuppressants, lipid-lowering agents, surgery, and radiotherapy, have shown limited and inconsistent benefit, with cladribine demonstrating the highest reported response rate in a recent review^2^

The Janus kinase (JAK) signal transducer and activator of transcription pathway is increasingly understood to be central to histiocytic proliferation and cytokine-driven inflammation. JAK inhibitors have demonstrated benefit in other histiocytic or related inflammatory syndromes, suggesting a potential role in XD.^3^ Upadacitinib, a selective JAK1 inhibitor currently approved for inflammatory conditions including atopic dermatitis and psoriatic arthritis, has not previously been studied in XD.^4^ Reviews and case series support the effectiveness of JAK inhibitors in other histiocytic and granulomatous disorders such as sarcoidosis and granuloma annulare.^5^ These observations suggest that cytokine signaling through JAK-signal transducer and activator of transcription is a unifying pathogenic axis across multiple histiocytic disorders and highlight its potential as a therapeutic target in XD.

In our patient, the addition of Upadacitinib to statin therapy resulted in a dose-dependent reduction of papules ultimately leading to complete clearance at 45 mg daily. Remarkably, disease remission was maintained during tapering and after discontinuation. This outcome supports the hypothesis that JAK1-driven cytokine signaling plays a possible role in XD pathogenesis and that selective inhibition may provide therapeutic benefit.

To our knowledge, this is the first reported case of XD successfully treated with upadacitinib, showing marked clinical improvement and good tolerability. This highlights upadacitinib as a promising therapeutic option and expands the potential role of JAK inhibitors in XD.

## Conflicts of interest

None disclosed.
